# Disseminated Endometriosis and Low-Grade Endometrioid Stromal Sarcoma in a Patient with a History of Uterine Morcellation for Adenomyosis

**DOI:** 10.1155/2020/7201930

**Published:** 2020-02-05

**Authors:** Daniel P. Stefanko, Ramez Eskander, Omonigho Aisagbonhi

**Affiliations:** ^1^Department of Pathology, University of California, San Diego, CA, USA; ^2^Department of Obstetrics, Gynecologic and Reproductive Sciences, Division of Gynecologic Oncology, University of California, San Diego, CA, USA

## Abstract

Morcellation of benign uterine tumors allows for removal of the tumors via minimally invasive laparoscopic procedures. However, in rare cases, morcellation has been associated with upstaging of unexpected malignancies. Morcellation has also been associated with dissemination of benign pathologic processes such as endometriosis and leiomyomas. Endometrial stromal sarcoma typically arises in the uterine cavity, although cases of extrauterine endometrioid stromal sarcoma arising out of foci of endometriosis have been reported. Dissemination of endometrial stromal sarcomas can be an unintended consequence of morcellation procedures, as can dissemination of endometriosis, from which endometrioid stromal sarcomas can arise. Herein, we report a case of a 55-year-old woman who was found to have disseminated endometriosis and low-grade endometrioid stromal sarcoma, with bowel and liver parenchymal metastasis, 7 years after undergoing supracervical hysterectomy with unconfined uterine morcellation for adenomyosis. Our case highlights the potential for malignant transformation of disseminated adenomyosis/endometriosis and the importance of patient counseling and shared decision-making prior to morcellation procedures.

## 1. Introduction

Adenomyosis and endometriosis define processes in which ectopic endometrial tissue is found in the myometrium or in extrauterine sites, respectively. Malignant transformation of endometriosis is estimated to occur in 1% of endometriosis cases with endometriosis being associated with extrauterine endometrioid and clear cell carcinomas as well as extrauterine adenosarcomas and endometrioid stromal sarcomas [1, 2]. Morcellation is a useful surgical technique that allows for the removal of uterine tumors via a minimally invasive laparoscopic approach. Morcellation is contraindicated in patients with known uterine malignancies. Numerous patients currently undergo morcellation for benign indications, predominantly leiomyomas. The risk for occult malignancies in these patients is low—ranging from 1 in 350 cases to 2 in 8720, depending on the study [3, 4]. However, power morcellation may also be associated with dissemination of endometriosis and other nonmalignant tumors and tumor-like conditions. Various studies have reported sequelae that include endometriosis, adenomyosis, and disseminated peritoneal leiomyomatosis following power morcellation for endometriomas, leiomyomas, or adenomyosis [5, 6].

Herein, we present a case of patient who developed disseminated endometriosis and endometrioid stromal sarcoma 7 years after undergoing unconfined uterine power morcellation for adenomyosis. Our case supports existing studies that show a potential for malignant transformation of endometriosis. We suggest appropriate patient counseling and consideration of alternatives to unconfined power morcellation in patients with endometriosis and/or adenomyosis.

## 2. Case Presentation

The patient was a 48-year-old, gravida 2, para 2 woman who initially presented to an outside hospital with heavy menstrual bleeding. Pelvic ultrasound revealed an 11 × 11 × 10 cm uterus with a 1.6 cm thick endometrial lining and multiple fibroids, the dominant one measuring 6 cm. Endometrial biopsy showed secretory endometrium without hyperplasia or neoplasia. She subsequently underwent laparoscopic supracervical hysterectomy with unconfined uterine morcellation, left salpingectomy, and appendectomy. Intraoperative findings were notable for a large uterus with a large fundal fibroid, left paratubal cyst, cecal adhesions with sclerosed appendiceal tip, normal ovaries, and grossly unremarkable liver and stomach. Gross pathologic evaluation at the outside facility showed a 475-gram, 24 × 17 × 6.5 cm morcellated fragmented uterus with numerous tan-white firm whorled myometrial nodules ranging from 0.2 cm to 9.5 cm in greatest dimension. No areas of hemorrhage or necrosis were grossly identified. Histologic assessment showed uterine adenomyosis, leiomyomas, and proliferative endometrium, fibrous obliteration of the appendiceal lumen and a benign left fallopian paratubal cyst.

**Figure 1:**
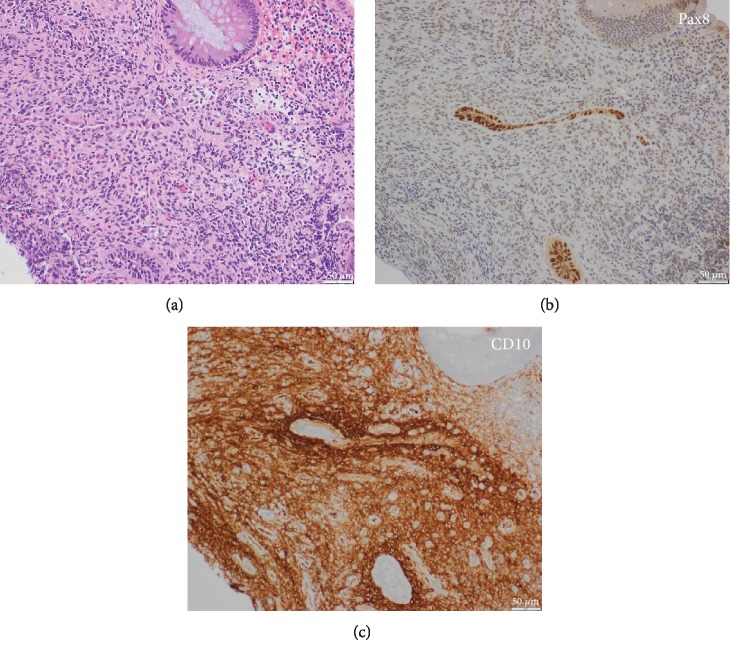
Colonic endometriosis with endometrial glands and stroma; hematoxylin and eosin stain (a). Pax8 highlights endometrial glands (b) while CD10 highlights endometrial stroma (c).

Four years after her surgical procedure, she developed constipation, bloody narrow caliber stools, and anemia and was found to have two extrinsic masses measuring 3 cm and 6 cm with features suggestive of erosion into the sigmoid colon on colonoscopy. Biopsy of the masses revealed endometriosis. Subsequent abdominal and pelvic MRI showed multiple soft tissue lesions throughout the abdomen and two liver lesions in segments 6 and 7, measuring 3.9 × 3.4 cm and 3.5 × 2.2 cm, respectively. The largest of the soft tissue lesions, measuring 4.9 × 4.5 cm, abutted the descending colon. FNA and core biopsies of the sigmoid colon and right perihepatic soft tissue lesions were consistent with endometriosis (Figure 1). She was started on an aromatase inhibitor, and 3- and 12-month follow-up MRI showed an interval decrease in the size of the intraperitoneal and hepatic lesions.

**Figure 2:**
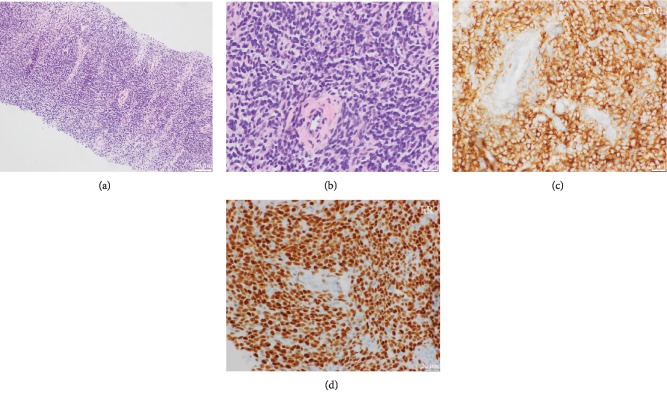
Biopsy showing low-grade endometrioid stromal sarcoma with diffuse endometrial stroma and spiral arterioles without glands; hematoxylin and eosin stain at 10x (a) and 40x (b) magnifications. The cells are diffusely CD10 (c) and ER (d) positive.

Over the following years, she maintained close clinical follow-up including yearly surveillance imaging. Seven years after initial surgery, her surveillance CT scan and subsequent pelvic MRI showed an interval increase in size of the known intraperitoneal lesions and numerous newly identified lesions, many of which demonstrated enhancement and diffusion restriction, concerning for malignant transformation. A biopsy was performed on one of the radiographically suspicious abdominal lesions. Microscopic examination revealed a low-grade endometrioid stromal sarcoma (Figure 2).

**Figure 3:**
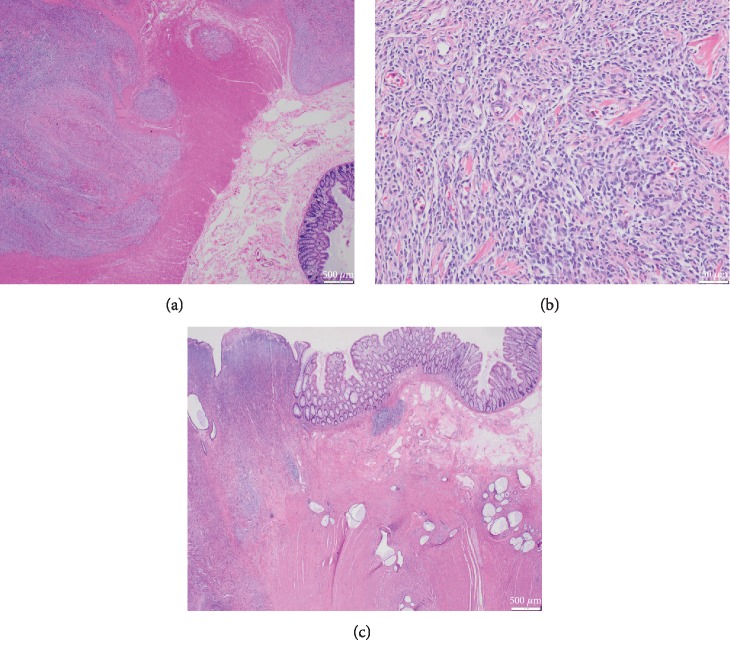
Resection specimen showing low-grade endometrioid stromal sarcoma with tongue-like infiltration of the colonic muscularis propria on low magnification (a), low-grade endometrial stroma and spiral arterioles on medium magnification (b), and background-associated endometriosis (c).

The patient underwent an extensive surgical cytoreduction with excision of multiple intraperitoneal lesions. The largest, measuring 15 cm, involved the proximal jejunum and a conglomerate of neighboring bowel. She additionally underwent a left oophorectomy, right salpingo-oophorectomy, omentectomy, three small bowel resections involving portions of the jejunum, proximal ileum and distal ileum, and resections of the segments 6 and 7 liver lesions via partial hepatectomy. Histopathological examination revealed low-grade endometrioid stromal sarcoma that diffusely involved the jejunum, proximal and distal ileum, sigmoid colon, liver segments 6 and 7, porta hepatis, and multiple soft tissue implants throughout the abdomen. There was notable associated endometriosis (Figure 3). The ovaries were uninvolved (Supplemental [Supplementary-material supplementary-material-1]). At completion of surgery, there was no visible residual intraperitoneal disease.

Following surgical resection, she was started on maintenance aromatase inhibitor therapy with oral letrozole, with continued follow-up over 9 months. Recent surveillance imaging showed no evidence of intraperitoneal disease recurrence, with a stable subcutaneous abdominal wall lesion.

## 3. Discussion

The concept of extrauterine malignancies arising from malignant transformation of ectopic endometrial tissue dates as far back as 1925 where Sampson made a case for endometrioid adenocarcinoma of the ovary arising out of endometriosis [7]. Since that time, various epidemiologic and histologic studies have shown a correlation between extrauterine endometrioid and clear cell adenocarcinomas and endometriosis. In a population-based cohort study of over 99,000 women in Denmark, Brinton et al. [8] observed relative risks of 2.53 and 3.37, respectively, for the subsequent development of ovarian endometrioid and clear cell carcinomas 5 or more years after the diagnosis of endometriosis. A nationwide 14-year historic cohort study of the Taiwanese National Health Insurance Research Database found a more dramatic 18.7-fold incidence rate of epithelial ovarian cancer in patients with a history of tissue-proven ovarian endometriomas compared to those without any diagnosis of endometriosis [9]. Recent molecular studies show identical *ARID1A* and *PIK3CA* mutations as well as loss of *PTEN* heterozygosity in endometriosis lesions adjacent to clear cell and endometrioid adenocarcinomas as well as in the carcinomas themselves [10, 11].

Extrauterine endometrioid stromal sarcomas are likewise thought to arise from malignant transformation of ectopic endometrial tissue. In their study of 27 cases of primary ovarian endometrial stromal sarcomas, Oliva et al. [12] found an intimate association with endometriosis in 16 of the 27 cases. Masand et al. [13] studied 63 cases of extrauterine endometrial stromal sarcomas with sites that included the ovaries, bowel wall, abdomen/peritoneum, pelvis, vagina, and cases involving multiple sites at the same time. They found associated endometriosis in 30 of the 63 cases. Multiple single case reports have also described low-grade endometrioid stromal sarcomas arising in association with concurrent endometriosis or following hysterectomies for adenomyosis or endometriosis with postsurgical time frame as long as 37 years [14]. Interestingly, similar *JAZF1-SUZ12*, *EPC1-PHF1* fusions, and *PHF1* rearrangements have been reported in both uterine and extrauterine endometrial stromal sarcomas, including those associated with endometriosis [15].

All together, the data suggest that endometriosis, though thought of as benign, may actually be a premalignant condition. Evidence to support this notion includes Lac et al.'s [16] finding of somatic cancer-driver hotspot mutations in *KRAS*, *ERBB2*, *PIK3CA*, and *CTNNB1* and heterozygous *PTEN* loss in archived endometriotic lesions.

In 2014, the FDA issued a safety communication discouraging the use of power morcellation for uterine fibroids [3]. This communication was issued due to concerns for the spread of unexpected sarcomas, estimated to occur at a rate of 1 in 350 patients undergoing hysterectomy or myomectomy for fibroids. In an open letter to the FDA published in 2016 [4], many leading gynecologists questioned the FDA's estimates of occult sarcomas suggesting much lower rates ranging from 1 in 1550 to 2 in 8720. This notable variation in the estimates of the incidence of occult sarcomas following surgery for benign gynecologic conditions appears to depend on the population studied and statistical methods used. Regardless of the true incidence of occult malignancies following surgery for benign gynecologic conditions, the use of power morcellation has been associated with the upstaging of the occult cancers, the consequences of which can be devastating for the individual patient. Emerging data suggests that, in addition to the potential for the spread of occult malignancies, unconfined morcellation may be associated with the dissemination of benign pathologic process such as endometriosis, adenomyosis, and leiomyomas [5, 6].

Our patient underwent unconfined power morcellation for leiomyomas and adenomyosis and subsequently developed disseminated endometriosis with malignant transformation into low-grade endometrioid stromal sarcoma. Malignant transformation of endometriosis most commonly affects the ovaries [2]. However, in our patient, the ovaries were uninvolved. Her involved sites included the gastrointestinal tract, liver, and peritoneum. The dissemination of endometriosis, a morbid condition that can be associated with disabling pain and heavy bleeding sometimes necessitating transfusions, along with the risk of malignant transformation, reflects significant reasons for gynecologic surgeons to consider alternatives to unconfined power morcellation for uterine mass lesions and to appropriately counsel patients regarding risk, even in the benign setting.

Although power morcellation is a valuable tool, allowing removal of uterine tumors via minimally invasive approaches, continued investigation into the implications of morcellation even in the context of benign conditions is required. Ultimately, given the exceedingly rare frequency of occult malignancy or malignant transformation of benign processes, balanced and comprehensive informed consent is required. Furthermore, alternatives to unconfined power morcellation which still permit minimally invasive laparoscopic surgery may be considered [17, 18].

## Supplementary Materials

Supplementary Materials Supplemental Figure: both ovaries were uninvolved. Sections of the left ovary revealed unremarkable ovarian stroma (A). The right ovary contained a 0.3 cm partially necrotic granuloma, but had no evidence of sarcoma; low magnification (B) and medium magnification (C).
