# Supplementary figures and images for: Disseminated Endometriosis and Low-Grade Endometrioid Stromal Sarcoma in a Patient with a History of Uterine Morcellation for Adenomyosis
Source: Case Rep Obstet Gynecol. 2020 Feb 5;2020:7201930. doi: 10.1155/2020/7201930 (PMC7025032; doi:10.1155/2020/7201930)

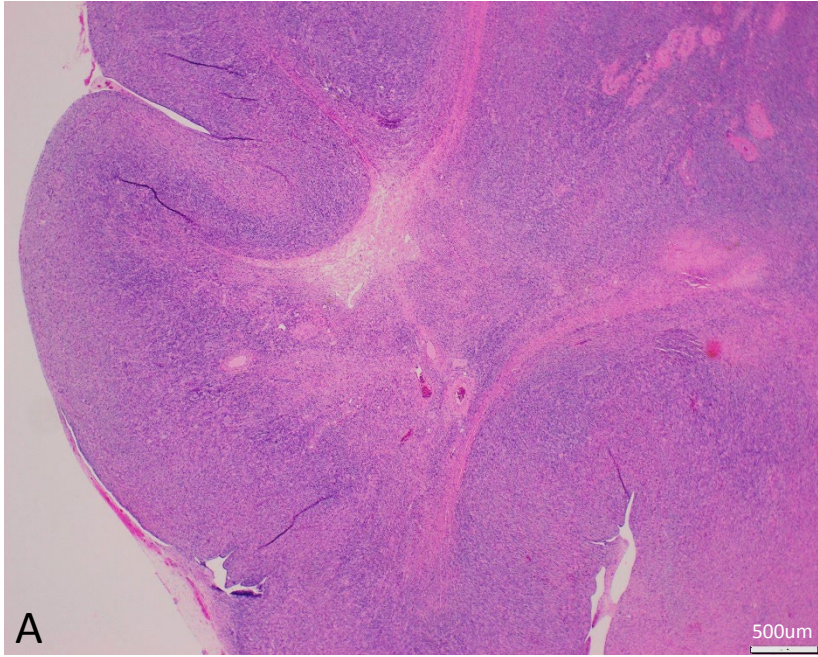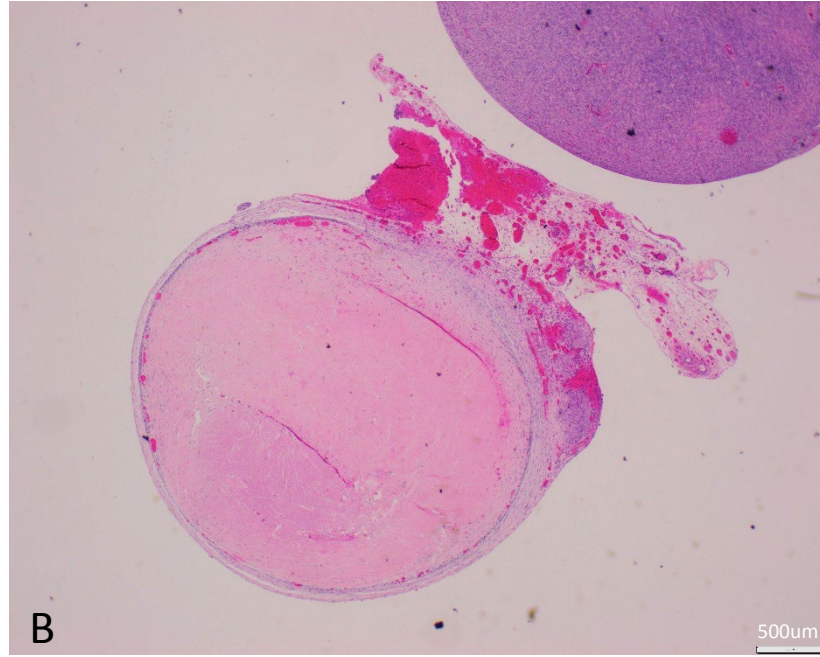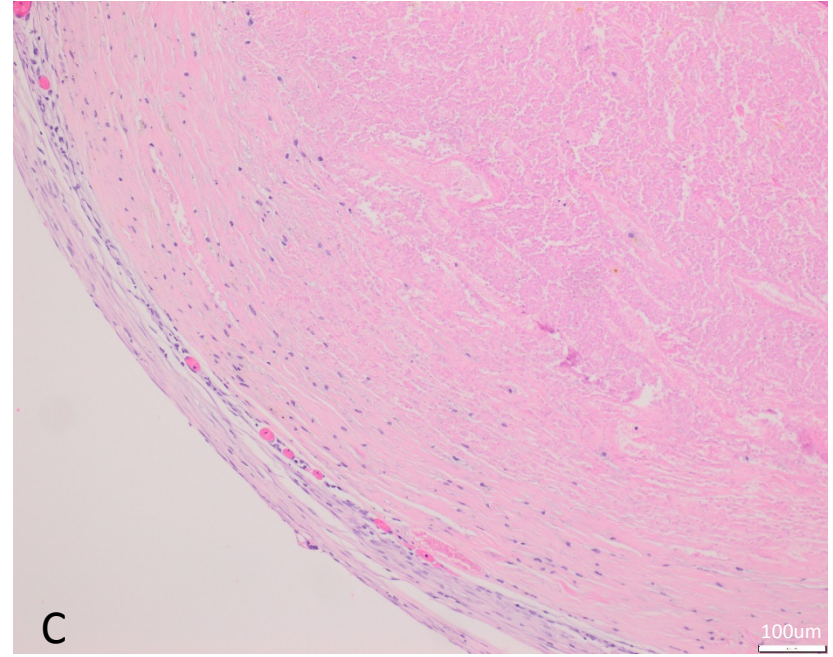

Supplemental Figure

Supplement: Supplementary Materials — Supplemental Figure: both ovaries were uninvolved. Sections of the left ovary revealed unremarkable ovarian stroma (A). The right ovary contained a 0.3 cm partially necrotic granuloma, but had no evidence of sarcoma; low magnification (B) and medium magnification (C). [file 7201930.f1.pdf]
